# A Nested–Nested Sparse Array Specially for Monostatic Colocated MIMO Radar with Increased Degree of Freedom

**DOI:** 10.3390/s23229230

**Published:** 2023-11-16

**Authors:** Ye Chen, Meng Yang, Jianfeng Li, Xiaofei Zhang

**Affiliations:** 1 College of Electronic Information Engineering, Nanjing University of Aeronautics and Astronautics, Nanjing 210000, China; amberchenye@126.com (Y.C.); yangmeng19861213@126.com (M.Y.); lijianfeng@nuaa.edu.cn (J.L.); 2 CSSC Intelligent Innovation Research Institute, Beijing 100044, China

**Keywords:** nested array, DOA estimation, monostatic MIMO radar, sum and difference co-array, degrees of freedom

## Abstract

This paper mainly investigates the problem of direction of arrival (DOA) estimation for a monostatic MIMO radar. Specifically, the proposed array, which is called a nested–nested sparse array (NNSA), is structurally composed of two nested subarrays, a NA with $N_{1}+N_{2}$ elements and a sparse NA, respectively, with $N_{3}+N_{4}$ elements. The design process of NNSA is optimized into two steps and presented in detail. Setting NNSA as transmitter/receiver arrays, we derive the closed-form expression of consecutive DOFs and calculate the mutual coupling coefficient. Eventually, extensive simulations are carried out and the results verify the superiority of the proposed array over the previous arrays in terms of consecutive DOFs, array aperture and mutual coupling effect.

## 1. Introduction

In recent years, multiple input–multiple output (MIMO) radar has raised significant attention in the field of signal processing, which can improve the spatial resolution and direction of arrival (DOA) performance, which benefits from increased degrees of freedom (DOFs) and reduced mutual coupling effect [1,2,3,4,5]. Various algorithms of DOA estimation have already been proposed for monostatic or bistatic MIMO radar [6,7]. For instance, estimation of signal parameters using the rotational invariance techniques (ESPRIT) [8,9] algorithm was proposed for DOA estimation; multiple signal classification (MUSIC) [10] algorithm was exploited for joint direction of departure (DOD) and DOA estimation in bistatic MIMO radar [11]. However, since the conventional MIMO radar usually exploits the uniform linear arrays (ULAs) in transmitter/receiver arrays, the inter-element spacing should not be greater than half of the wavelength. Under the condition of finite physical sensors, it stands to reason that both the array aperture and the attainable DOFs are limited.

To obtain increased DOFs and improve the DOA estimation performance, sparse arrays with reduced mutual coupling and greater DOFs have been proposed and exploited in MIMO radars. In [12], sparse nonuniform linear arrays (NLA) based on MIMO radars was exploited for better DOA estimation performance. The prototype coprime array (CPA) was exploited in transmitter/receiver arrays, enhancing the DOA estimation performance with more DOFs [13,14]. In [15], a pair of coprime ULAs was utilized in the MIMO radar framework, in which a sum co-array viewpoint was introduced and could achieve a better DOA estimation performance. In [16], the minimum redundancy array (MRA) [17] was exploited in MIMO radar, which could obtain $O(N^{2})$ DOFs with only O(N) physical sensors. However, the specified configuration of MRA requires an exhaustive search and there is no closed-form expression for consecutive DOFs from the physical position set. Moreover, a nested MIMO array structure was proposed in [18] by assembling NA [19] in transmitter/receiver arrays.

Compared to coprime MIMO radar, nested MIMO radar can produce a hole-free ULA in virtual array, which is desirable. NA has a dense subarray, in which the closely located sensors induce a severe mutual coupling effect and can decrease the DOA estimation performance significantly [20,21], while CPA suffers less from the mutual coupling effect due to a sparser element distribution. Nevertheless, coprime MIMO radar produces holes in its sum-difference co-array, which decreases the consecutive DOFs. In response to these issues, some improved arrays based on NA or CPA with better properties have been proposed. Augmented coprime array (ACA) in [22] was proposed to obtain more consecutive DOFs than CPA in second-order difference co-array (2-DC). Unfolded co-prime linear array (UCLA) [23] was constructed by flipping one subarray along the origin to reduce the redundancy of 2-DC. The aforementioned arrays are mainly designed based on 2-DC. After introducing fourth-order difference co-array (4-DC), fourth-level nested array (FL-NA) [24] and three-level nested array (THRL-NA) [25] have been proposed to obtain more DOFs and less redundancy than NA.

Considering that the process of obtaining 4-DC from physical sensors is equivalent to obtaining second-order difference co-array of sum co-array (2-DCSC), it provides a prospective design for a MIMO array configuration. Thus, the design of a MIMO array configuration can be regarded as solving the problem of obtaining 2-DCSC of a concrete array. Guiding by this view, we propose a nested MIMO array configuration in this paper.

The main contributions of this paper are briefly summarized as follows:

1. We propose a sparse MIMO array configuration called NNSA, which is composed of two subarrays: a NA and a sparse NA, respectively. The basic idea of designing NNSA is based on the property of NA.
2. Considering that it is complicated to obtain a consecutive 2-DCSC from physical sensors directly, we optimize the design process by simplifying it into two steps: extracting the consecutive DOFs in 2-SC from physical sensors and subsequently calculating the 2-DC of 2-SC to obtain a consecutive virtual 2-DCSC as long as possible. This step-by-step simplification enhances the efficiency of designing NNSA. Moreover, given the total number of physical sensors *T*, it is specified how to select $N_{1}$, $N_{2}$, $N_{3}$, and $N_{4}$ to accomplish the maximal consecutive DOFs.
3. Comparing NNSA with other arrays, we assess the ability of NNSA in DOA estimation. The simulation results confirm the superior properties of NNSA. The proposed NNSA enjoys increased consecutive DOFs, larger array aperture, weaker mutual coupling effect and smaller error in DOA estimation.

In this paper, lower-case and upper-case bold characters represent the vectors and matrices, respectively. $E\left\{\cdot \right\}$ stands for the expectation of a random variable and ${∥\cdot ∥}_{F}$ indicates the Frobenius norm. The superscripts ${\left(\cdot \right)}^{T}$, ${\left(\cdot \right)}^{*}$, ${\left(\cdot \right)}^{H}$ and ${\left(\cdot \right)}^{-1}$ are the transpose, conjugate, Hermitian-transpose, and inversion, respectively. ${\langle S\rangle }_{i}$ refers to the i-th element in *S*. ⊗ and ∘ refers to the Kronecker and Khatri-Rao product. vec(·) represents the vectorization operator. $\mathrm{diag}\left\{\cdot \right\}$ implies a diagonal matrix.

## 2. Preliminaries

This section begins with the definitions of 2-DC, 2-SC, 4-DC, 2-DCSC and signal model for monostatic colocated MIMO radar.

### 2.1. Related Definitions

Given a linear array with *M* sensors and the unit inter-element spacing *d*, the position set can be described as follows [26]:

(1) $$S=\{p_{1}d,p_{2}d,\ldots ,p_{M}d\},i=1,2,\ldots ,M$$

where $p_{i}d$ denotes the position of the $p_{i}$-th sensor and d=λ/2.

According to (1), several definitions are introduced for a given linear array with position set *S*:

**Definition** **1.** *The second-order difference co-array $S_{2-DC}$ is defined as [27]*

(2) $$S_{2-DC}=\left\{\left(p_{i}-p_{j}\right)d,1\leq i,j\leq M\right\}$$

**Definition** **2.** *The second-order sum co-array $S_{2-SC}$ is defined as [28]*

(3) $$S_{2-SC}=\left\{\left(p_{i}+p_{j}\right)d,1\leq i,j\leq M\right\}$$

**Definition** **3.** *The fourth-order difference co-array $S_{4-DC}$ is defined as [29]*

(4) $$S_{4-DC}=\left\{\left(\left(p_{i}-p_{k}\right)-\left(p_{j}-p_{l}\right)\right)d,1\leq i,j,k,l\leq M\right\}$$

**Definition** **4.** *The second-order difference co-array of sum co-array $S_{2-DCSC}$ is defined as [12]*

(5) $$\begin{array}{cc} S_{2-DCSC} & =\{\left(\left(p_{i}+p_{j}\right)-\left(p_{k}+p_{l}\right)\right)d,1\leq i,j,k,l\leq M\}\\ \; & =\{\left(\left(p_{i}-p_{k}\right)-\left(p_{j}-p_{l}\right)\right)d,1\leq i,j,k,l\leq M\}\\ \; & =S_{4-DC} \end{array}$$

### 2.2. Signal Model

Assume that a monostatic MIMO radar consists of a transmitter/receiver array with *M* and *N* elements respectively, the position set of which is depicted in (1), with *K* far-field narrowband sources impinging on the receiver array, from directions $\theta =[\theta_{1},\theta_{2},\ldots ,\theta_{K}]$. The observed output signal of matched filters can be modeled as [30]

(6) $$x\left(t\right)=\left(A_{r}\circ A_{t}\right)s\left(t\right)+n\left(t\right)=As\left(t\right)+n\left(t\right)$$

where $A_{t}=\left[a_{t}\left(\theta_{1}\right),a_{t}\left(\theta_{2}\right),\ldots ,a_{t}\left(\theta_{K}\right)\right]$ denotes the transmitting direction matrix while $A_{r}=\left[a_{r}\left(\theta_{1}\right),a_{r}\left(\theta_{2}\right),\ldots ,a_{r}\left(\theta_{K}\right)\right]$ denotes the receiving direction matrix. The steering vectors are represented by $a_{t}\left(\theta_{k}\right)$ and $a_{r}\left(\theta_{k}\right)$.

(7) $$a_{t}\left(\theta_{k}\right)={\left[e^{-j2\pi p_{1}^{t}dsin\left(\theta_{k}\right)/\lambda },\ldots ,e^{-j2\pi p_{M}^{t}dsin\left(\theta_{k}\right)/\lambda }\right]}^{T}\in C^{M\times 1}$$

(8) $$a_{r}\left(\theta_{k}\right)={\left[e^{-j2\pi p_{1}^{r}dsin\left(\theta_{k}\right)/\lambda },\ldots ,e^{-j2\pi p_{N}^{r}dsin\left(\theta_{k}\right)/\lambda }\right]}^{T}\in C^{N\times 1}$$

And $s\left(t\right)={\left[s_{1}\left(t\right),s_{2}\left(t\right),\ldots ,s_{K}\left(t\right)\right]}^{T}\in C^{K\times 1}$, $s_{k}\left(t\right)$ represents source signal of the *k*-th target. In this paper, under the background of monostatic colocated MIMO radar, we exploit the same array as both the transmitter/receiver array and thus $A_{r}=A_{t}$. Besides, n(t) represents the received Gaussian white noise with mean zero and variance $\sigma^{2}$, $n\left(t\right)∼N\left(0,\sigma^{2}\right)$. The covariance matrix of received signals is given by

(9) $$R=E\left[x\left(t\right)x^{H}\left(t\right)\right]=AR_{s}A^{H}+\sigma^{2}I_{M\times M}$$

where $R_{s}=E\left[s\left(t\right)s^{H}\left(t\right)\right]=diag\left\{\left[\sigma_{1}^{2},\sigma_{2}^{2},\ldots ,\sigma_{K}^{2}\right]\right\}$ represents the signal covariance matrix while $\sigma_{k}^{2}$ denotes the signal power of *k*-th target. In practice, the covariance matrix of received signals is estimated by *L* samplings (t=1,2,…,L), i.e.,

(10) $$\hat{R}=\frac{1}{L}\sum_{t=1}^{L}x\left(t\right)x^{H}\left(t\right)$$

By vectorizing R [31],

(11) $$z=vec\left(R\right)=\left(A^{*}\circ A\right)p+\sigma^{2}vec\left(I\right)$$

where $p={\left[\sigma_{1}^{2},\sigma_{2}^{2},\ldots ,\sigma_{K}^{2}\right]}^{T}$. From (11), we can calculate the consecutive DOFs of the proposed array. Despite the above discussion based on the absence of mutual coupling, it is necessary to take the mutual coupling effect into account in practical scenarios. The signal output of each sensor is influenced by adjacent elements and the output signals of matched filters can be formulated as [32]

(12) x(t)=CAs(t)+n(t)

where C represents the mutual coupling matrix, which can be expressed by [32]

(13) $$C_{i,j}=\left\{\begin{array}{cc} 0, & |{\langle S\rangle }_{i}-{\langle S\rangle }_{j}|>B\\ c_{|{\langle S\rangle }_{i}-{\langle S\rangle }_{j}|}, & |{\langle S\rangle }_{i}-{\langle S\rangle }_{i}|\leq B \end{array}\right.$$

where $s_{i},s_{j}\in S$ and the mutual coupling coefficients satisfy $1=c_{0}>|c_{1}\left|>\ldots >\right|c_{B}\left|>\right|c_{B+1}|$, $c_{1}=0.5e^{j\pi /4}$, $c_{k}=c_{1}e^{\left(-j(k-1)\pi /8\right)}$, $|c_{B+1}|=0$, k=2,3,…,B, in this paper B=100.

Furthermore, in a specific array, the mutual coupling coefficient can be quantified by a parameter named coupling leakage [33].

(14) $$L\left(M\right)=\frac{{\left\|C-diag\left\{C\right\}\right\|}_{F}}{{\left\|C\right\|}_{F}}$$

From (13), the received signal of virtual array with mutual coupling in (11) can be derived as

(15) $$\tilde{z}=C_{vec}\left(A^{*}\circ A\right)p$$

where $C_{vec}={\left(C⊗C^{*}\right)}^{*}⊗\left(C⊗C^{*}\right)$.

## 3. Proposed Array Configuration

The design process of the proposed array is discussed in this section, as well as a specific example.

### 3.1. Design of the Proposed Array

**Figure 1:**
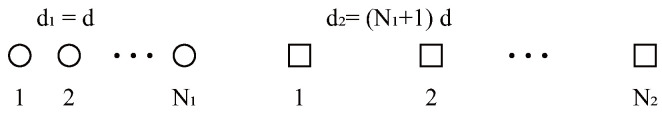
The structure of nested array.

Figure 1 illustrates the structure of NA, which consists of two ULA subarrays. Subarray1 is composed of $N_{1}$ elements with inter-element spacing of *d* while subarray2 consists of $N_{2}$ elements with inter-element spacing of $\left(N_{1}+1\right)$d. It has been confirmed in [19] that the 2-DC of NA is continuous with $2N_{2}\left(N_{1}+1\right)-1$ consecutive DOFs. This serves as a crucial foundation of the proposed array.

In this paper, by means of utilizing the property of NA, we provide a simplified approach to designing the proposed array. The key to the array design is exploiting the consecutive part from the 2-SC of the two subarrays and obtaining a virtual NA that is as long as possible.

**Figure 2:**
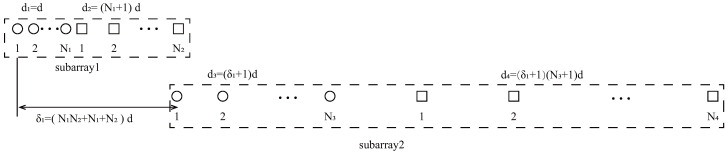
The configuration of the proposed array.

Figure 2 illustrates the structure of NNSA. Given a specific number of sensors *T*, NNSA is composed of two subarrays: a prototype NA and a sparse NA, respectively. According to [19], subarray1, a NA, contains two ULAs. Among the two ULAs, the first ULA has $N_{1}$ sensors with inter-element spacing *d*, while the second ULA starts at $N_{1}d$ and has $N_{2}$ sensors with inter-element spacing $(N_{1}+1)d$. Thus, the position set of physical sensors of the first NA can be denoted as

(16) $$\begin{array}{c} S=\left\{n_{1}d,1\leq n_{1}\leq N_{1}-1\right\}\cup \left\{N_{1}d+n_{2}\left(N_{1}+1\right)d,1\leq n_{2}\leq N_{2}-1\right\} \end{array}$$

the DOFs of 2-DCSC can also be calculated: $2(N_{1}N_{2}+N_{1}+N_{2})-1$. Set the starting point of subarray2 at $\delta_{1}=\left(N_{1}N_{2}+N_{1}+N_{2}\right)d$. Likewise, the second subarray has two sparse ULAs. The first sparse ULA has $N_{3}$ sensors with inter-element spacing $(\delta_{1}+1)d$, while the second sparse ULA starts at $\delta_{1}d$ and has $N_{4}$ sensors with inter-element spacing $\left(\delta_{1}+1\right)\left(N_{3}+1\right)d$, respectively. Thus, the position of physical sensors in NNSA can be represented as

(17) $$S=S_{1}\cup S_{2}$$

(18) $$\begin{array}{c} S_{1}=\left\{n_{11}d,0\leq n_{11}\leq N_{1}-1\right\}\cup \left\{N_{1}d+n_{12}\left(N_{1}+1\right)d,0\leq n_{12}\leq N_{2}-1\right\} \end{array}$$

(19) $$\begin{array}{cc} S_{2}=\delta_{1}d+\left\{n_{21}\left(\delta_{1}+1\right)d\right\}\cup \left\{N_{1}\left(\delta_{1}+1\right)d+n_{22}\left(\delta_{1}+1\right)\left(N_{3}+1\right)d\right\} & \\ 0\leq n_{21}\leq N_{3}-1,0\leq n_{22}\leq N_{4}-1 \end{array}$$

Given the number of total physical sensors *T*, to simplify the calculation, we set $N_{1}=N_{3},N_{2}=N_{4}$ and $T=\sum_{i=1}^{4}N_{i}=2\left(N_{1}+N_{2}\right)$. According to [19], the number of consecutive DOFs of 2-SC in subarray1 is $N_{2}\left(N_{1}+1\right)+N_{1}$, which is denoted as $\delta_{1}$. Because of the same geometry of the two subarrays, the number of consecutive DOFs of 2-SC in subarray2 is also $\delta_{2}$, $\delta_{1}=\delta_{2}$. In next section, we derive the closed-form expression of consecutive DOFs of 2-DCSC. Extracting the consecutive part from the two 2-SC, its position can be denoted as

(20) $$\begin{array}{c} S_{c2sc}=\left\{m_{1}d,0\leq m_{1}\leq \delta_{1}\right\}\cup \left\{2\delta_{1}d+m_{2}\left(\delta_{1}+1\right)d,0\leq m_{2}\leq \delta_{2}-1\right\} \end{array}$$

According to (20), the 2-DC of $S_{c2sc}$ is confirmed to be a consecutive virtual array, denoted as $S_{c2dcsc}$. It seems that there is a hole at the position of δd, since δ cannot be obtained based on the difference of any two elements in $S_{c2sc}$. Actually, δ is the difference of elements 0 and δ in the physical position set. Therefore, the position of $S_{c2dcsc}$ can be represented as

(21) $$\begin{array}{c} S_{c2dcsc}=\left\{-\left[\left(\delta_{1}+1\right)\left(\delta_{2}+1\right)-2\right]d,\ldots ,-2d,-d,0,d,2d,\ldots ,\left[\left(\delta_{1}+1\right)\left(\delta_{2}+1\right)-2\right]d\right\} \end{array}$$

According to (21), the maximal consecutive DOFs can be concluded

(22) $$\begin{array}{c} \mathrm{DOF}=2\left[\left(\delta_{1}+1\right)\left(\delta_{2}+1\right)-2\right]+1=2\delta_{1}\delta_{2}+2\delta_{1}+2\delta_{2}-1 \end{array}$$

### 3.2. A Specific Example of NNSA

**Figure 3:**
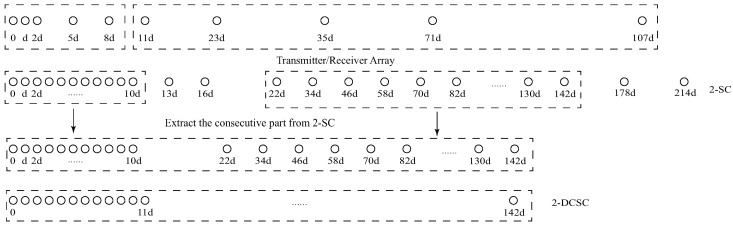
A specific example of the proposed array configuration.

From the aforementioned analysis, a specific example is given in Figure 3 to vividly verify the related conclusions. Suppose the number of physical sensors in two subarrays are $N_{1}=N_{3}=2,N_{2}=N_{4}=3$, respectively. Thus, the total number of physical sensors is $T=2(N_{1}+N_{2})=10$. The structure of the proposed array is composed of two parts: subarray1 is a NA with position set denoted as {0,1,2,5,8}d while subarray2 is a sparse NA, the position set of which is {11,23,35,71,107}d. Therefore, we can obtain the position set of the example, which can be represented as S={0,1,2,5,8,11,23,35,71,107}d. First, we obtain the 2-SC of the two subarrays, respectively, and secondly, we extract the consecutive part of the two 2-SC. The consecutive part of 2-SC position set of the first subarray is {0,1,2,3,4,5,6,7,8,9,10}d and the position set of the second subarray is {22,34,46,58,70,82,94,106,118,130,142}d. The combined two subarrays can be regarded as a new virtual sparse NA. Thus, based on Equation (22), the consecutive DOFs in 2-DCSC can be computed as 285.

Generally, it is specified how to select $N_{1}$, $N_{2}$, $N_{3}$ and $N_{4}$ to accomplish the maximal number of consecutive DOFs. In NNSA, the number of total physical sensors *T* should be set as an even number and the choice of $N_{1}$, $N_{2}$, $N_{3}$ and $N_{4}$ is given as shown below:

(23) $$\left\{\begin{array}{ccc} N_{1}=N_{2}=N_{3}=N_{4}=q; & \; & T=4q,q\in Z\\ N_{1}=N_{3}=q;\;N_{2}=N_{4}=q+1; & \; & T=4q+2,\in Z \end{array}\right.$$

### 3.3. Design Procedures

The design procedure of the proposed array can be outlined into two steps:

**Step 1:** Given the number *T*, the respective number of elements for two subarrays is set as $N_{1}$, $N_{2}$ and $N_{3}$, $N_{4}$ ($N_{1}$ = $N_{3}$, $N_{2}$ = $N_{4}$). The two position sets are denoted in (18) and (19);

**Step 2:** Acquire 2-SC from physical sensors; extract the consecutive part from 2-SC and subsequently calculate 2-DC of the consecutive part. The element set of 2-SC is shown in (20). Eventually, a consecutive 2-DCSC is attained by leveraging the consecutive property of 2-DC in NA, the position set of which is represented as (22). Hence, through progressive simplification, the process of attaining a consecutive 2-DCSC is streamlined as above.

## 4. Performance Comparison

In this section, we assess the performance of NNSA by conducting a comparative analysis with several arrays in the scenario of a monostatic colocated MIMO radar. The evaluation is based on a range of performance indices, including the attainable consecutive DOFs, the closed-form expression of consecutive DOFs from the number of physical sensors, a mutual coupling coefficient and redundancy.

The closed-form expressions for consecutive DOFs are listed in Table 1.

In Table 2, the normalized position of physical sensors, the redundancy distribution diagram of 2-SC and 2-DCSC, consecutive DOFs, SS-MUSIC spectrum and mutual coupling coefficient L(M) are provided. The total number of physical sensors is set as 10. $A_{r}=A_{t}$. Letting the position set of physical sensors for each array divided by inter-element spacing *d*, the normalized position is obtained. The redundancy is measured in terms of weight function; more specifically, the times of each element’s appearance at the corresponding point. In SS-MUSIC spectrum, there are 27 sources impinging on the arrays from direction [−39°,−36°,…,0,3°,…39°]. SNR = −5 dB and the number of snapshots is set as 100. We evaluate the ability to distinguish multiple targets of each array from peaks in spectrums.

Redundancy diagrams depict the times of the elements’ appearance in the corresponding positions. The graphs of 2-SC and 2-DCSC clearly illustrate that the proposed array configuration enjoys a broader distribution, except for UCLA, indicating reduced redundancy. From SS-MUSIC spectrum, it is confirmed that the spatial peaks of the proposed array are sharper and all the targets can be detected. In comparison, the mutual coupling coefficient of the proposed array turns out to be slightly greater than UCLA but smaller than ACA, NA, FL-NA and THRL-NA. A smaller mutual coupling coefficient indicates a weaker mutual coupling effect, leading to improved DOA estimation performance. Moreover, according to Table 1, the consecutive DOFs of ACA, NA, UCLA, FL-NA, THRL-NA and the proposed NNSA can reach 85, 117, 157, 215, 253 and 285 respectively. As illustrated above, the proposed array NNSA can obtain the greatest consecutive DOFs with 10 sensors and outperforms other arrays in terms of DOA estimation. Further simulations are carried out in the subsequent section.

## 5. Simulations Results

Relevant simulations are carried out in this section to validate the superior properties of the proposed array through Root Mean Square Error (RMSE) with 500 Monte Carlo experiments. The definition of RMSE is defined below [34]:

(24) $$RMSE=\sqrt{\frac{1}{500K}\sum_{i=1}^{500}\sum_{k=1}^{K}{\left(\theta_{k,i}-\theta_{k}\right)}^{2}}$$

where $\theta_{k}$ denotes the real angle of the *k*-th target while $\theta_{k,i}$ denotes the estimated angle of direction of the *k*-th target in the *i*-th experiment. Suppose the total number of sensors is 10 and there are 2 sources, θ=[10°,30°], impinging on the monostatic colocated MIMO radars—ACA, NA, UCLA, FL-NA, THRL-NA and the proposed NNSA included. $A_{r}=A_{t}$. Simulation experiments are conducted using the SS-ESPRIT algorithm in subsections *A*, *B*, *C* and *D*.

### 5.1. RSME Performance of Different Number of Sensors

**Figure 4:**
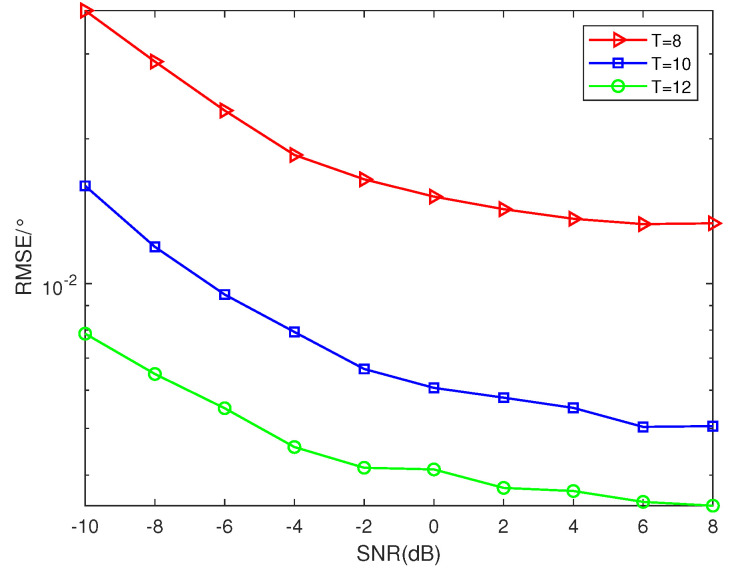
RMSE performance of different numbers of sensors (*K* = 2, SNR = 0 dB, *L* = 100).

Suppose that there are 2 sources *K* = 2 from the direction θ=[10°,30°], SNR = 0 dB. $A_{r}=A_{t}$. Figure 4 depicts three RMSE curves for different numbers of sensors. In this subsection, SNR varies from −10 dB to 8 dB and *L* is set as 100. It can be observed from Figure 4 that the number of sensors has an impact on the performance of DOA estimation: as the number of sensors increases, the proposed NNSA monostatic colocated MIMO radar exhibits improved accuracy in RMSE.

### 5.2. RSME Performance of Different Number of Snapshots

**Figure 5:**
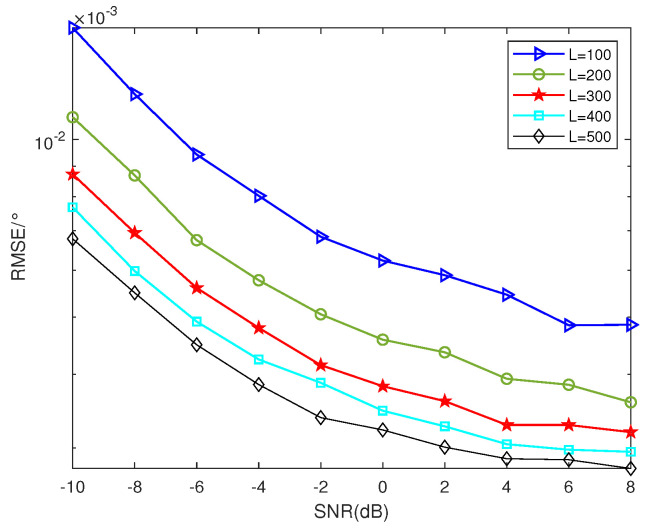
RMSE performance of different number of snapshots (*K* = 2, *T* = 10).

In this subsection, assuming that there are 2 sources *K* = 2 from the direction θ=[10°,30°], *T* = 10. $A_{r}=A_{t}$. As illustrated in Figure 5, the RMSE diminishes as the number of snapshots increases, thereby enhancing the performance of DOA estimation. The outcome can be interpreted that more snapshots lead to the presence of more samples in the signal, resulting in better DOA estimation accuracy. However, considering the saturation of samples, the extent of improvement is not unlimited and diminishes as the same amount of snapshots increases.

### 5.3. RSME Comparison of Different Arrays versus SNR

**Figure 6:**
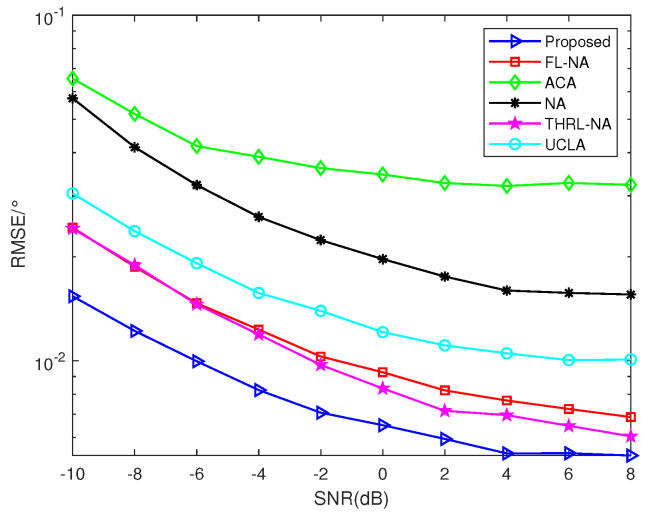
RMSE comparison of different arrays versus SNR (*K* = 2, *T* = 10, *L* = 100).

To vividly illustrate the superior property of NNSA monostatic colocated MIMO radar for DOA estimation, we statistically compare it with several other arrays: ACA, NA, UCLA, FL-NA and THRL-NA. Simulations are conducted under the conditions that $A_{r}=A_{t}$, θ=[10°,30°], *K* = 2, *T* = 10 and *L* = 100. As depicted in Figure 6, there are five RMSE curves of DOA estimation versus SNR. It is clear that the proposed array NNSA outperforms other arrays and enjoys smaller RMSE because of the greater consecutive DOFs and larger array aperture, verifying its superior property.

### 5.4. RSME Comparison of Different Arrays versus Snapshots

**Figure 7:**
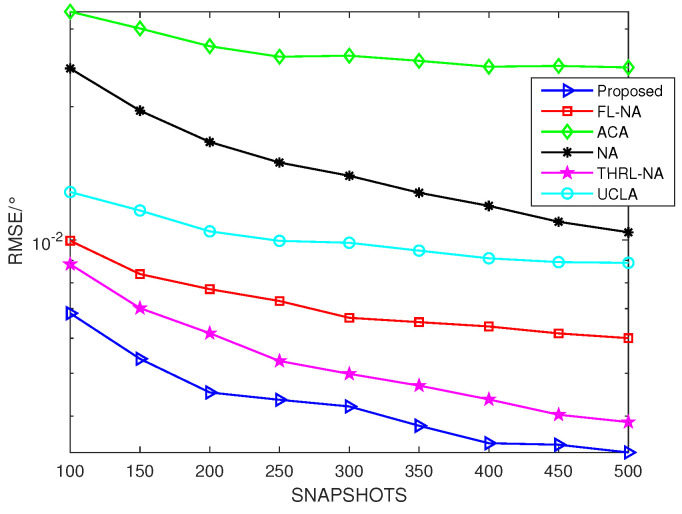
RMSE comparison of different arrays versus snapshots (*K* = 2, *T* = 10, SNR = 0 dB).

Assume that θ=[10°,30°], *K* = 2, *T* = 10, SNR = 0 dB and $A_{r}=A_{t}$ in this subsection. From Figure 7, it can be concluded that benefiting from greater consecutive DOFs and larger array aperture, the proposed NNSA monostatic colocated MIMO radar is able to accomplish smaller RMSE than ACA, NA, UCLA, FL-NA and THRL-NA. The simulation outcomes have verified its superior property.

## 6. Conclusions

In this paper, we propose an array configuration specially for monostatic colocated MIMO radar. This configuration, which is called NNSA, combines a NA and a sparse NA. Through offering a specific example, the design procedure involves two steps: acquiring 2-SC from physical sensors and subsequently calculating the 2-DC of the 2-SC. By extracting the consecutive part in 2-SC from physical sensors, we can obtain a consecutive virtual 2-DCSC with increased DOFs. Given the total number of physical sensors *T*, it is specified how to select $N_{1}$, $N_{2}$, $N_{3}$, and $N_{4}$ to accomplish the maximal consecutive DOFs. Additionally, we derive the closed-form expression of consecutive DOFs from physical sensors and it turns out that NNSA has increased consecutive DOFs compared to other arrays. In comparison, the proposed NNSA enjoys advantages over consecutive DOFs, SS-MUSIC spectrum and mutual coupling coefficient L(M). Monte Carlo experiments have been conducted and the results strongly indicate that compared with ACA, NA, UCLA, FL-NA and THRL-NA, NNSA obtains smaller RMSE in DOA estimation, confirming its superiority. In the future, it is significant to advance the work in nested MIMO array design based on NA if the 2-SC of physical sensors can satisfy the condition of optimal NA.

## Figures and Tables

**Table 1:** The closed-form expression for consecutive DOFs via the number of total sensors.

Arrays	Total Number of Sensors	Consecutive DOFs
	**(**$N_{i}$**,** ***i*** ** = 1, 2, …, 4)**	**(**$N_{i}$**,** ***i*** ** = 1, 2, …, 4)**
ACA	$2N_{1}+N_{2}-1$	$6N_{1}N_{2}+2N_{1}-2N_{2}-1$
NA	$N_{1}+N_{2}$	$4N_{2}\left(N_{1}+1\right)-3$
UCLA	$N_{1}+N_{2}$	$4N_{1}N_{2}-1$
FL-NA	$N_{1}+N_{2}+N_{3}+N_{4}-3$	$2N_{1}N_{2}N_{3}N_{4}-1$
THRL-NA	$N_{1}+N_{2}+N_{3}$	$4N_{3}\left(N_{2}+1\right)\left(N_{1}+1\right)-3$
Proposed	$2(N_{1}+N_{2})$	$2{\left(N_{1}N_{2}+N_{2}+N_{1}+1\right)}^{2}-3$

**Table 2:** The performance comparison with different arrays.

Arrays	ACA	NA	UCLA
Normalized position	{0, 3, 5, 6, 9, 10, 12, 15, 20, 25}	{1, 2, 3, 4, 5, 6, 12, 18, 24, 30}	{−25, −20, −15, −10, −5, 0, 6, 12, 18, 24}
2-SC	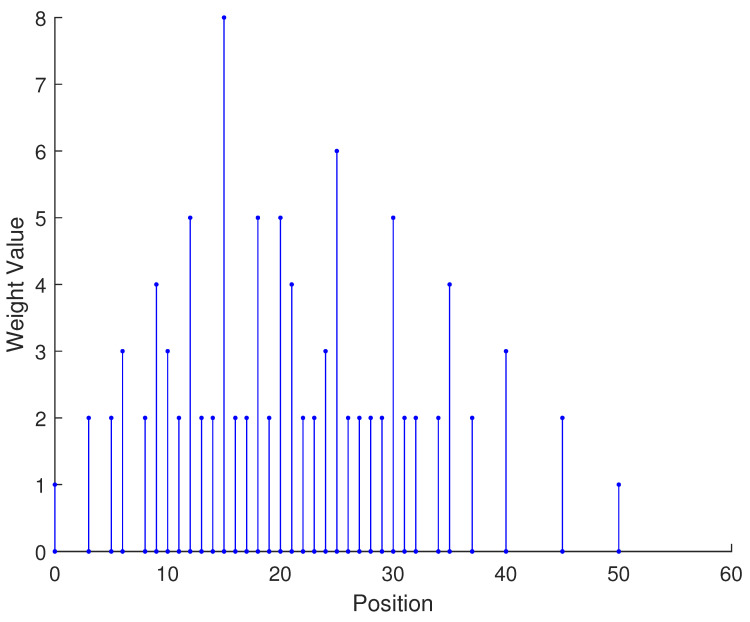	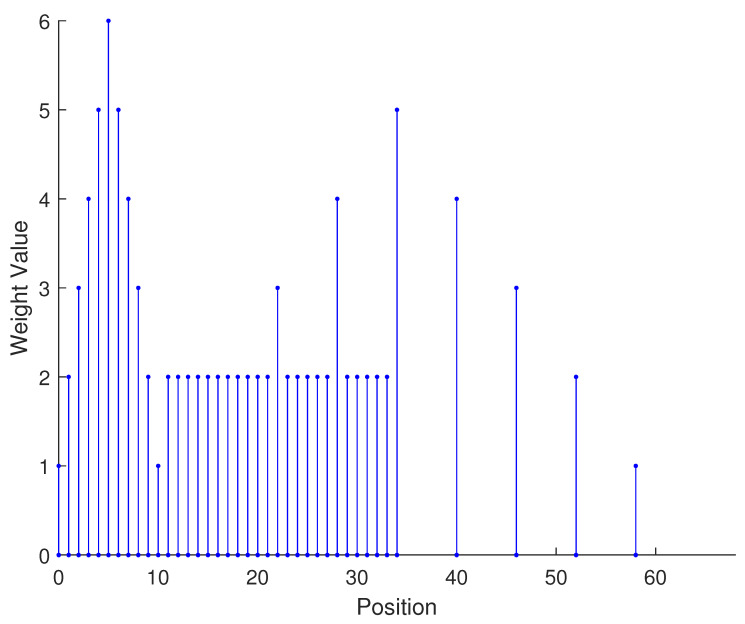	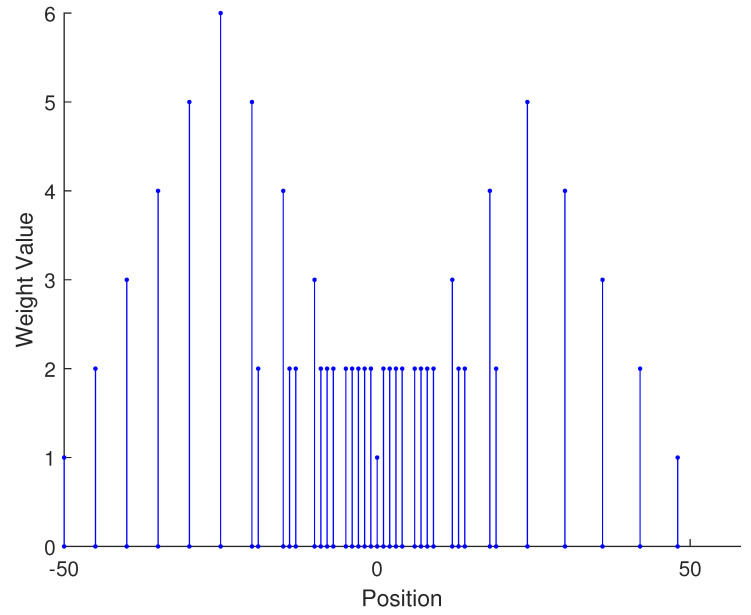
2-DCSC	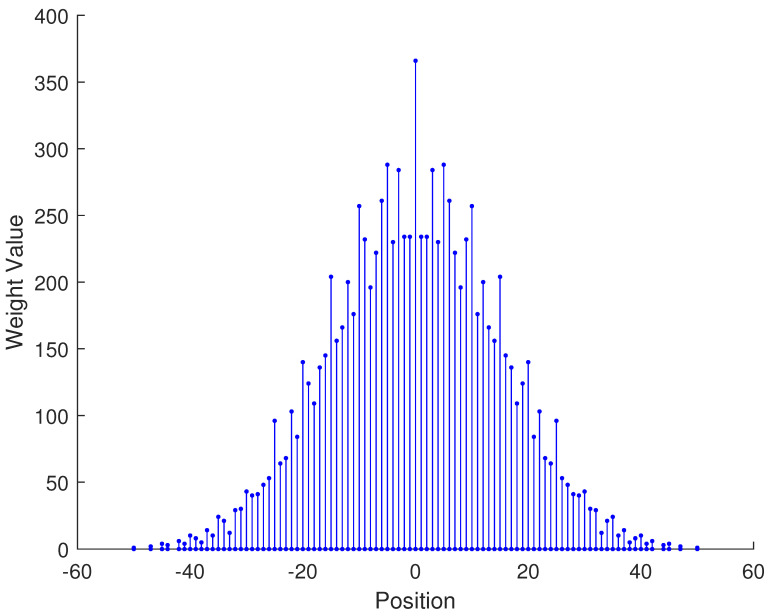	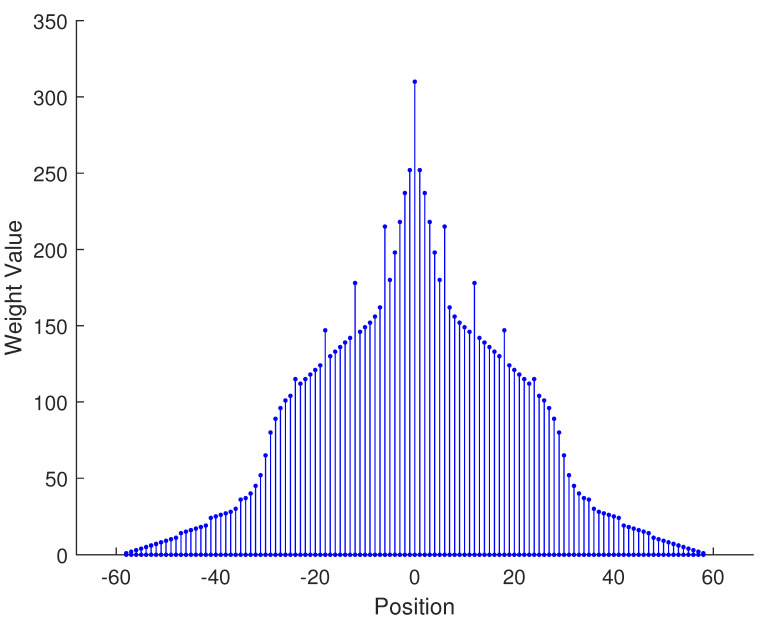	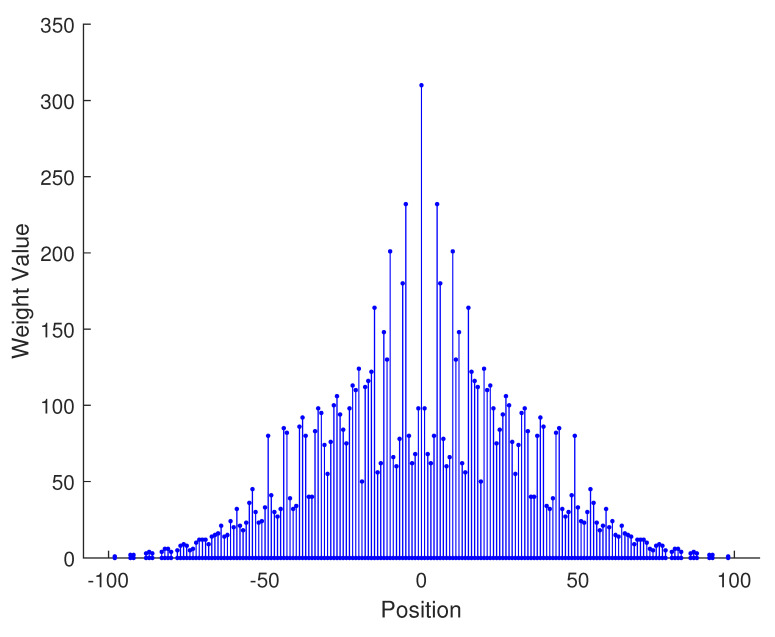
SS-MUSIC Spectrum	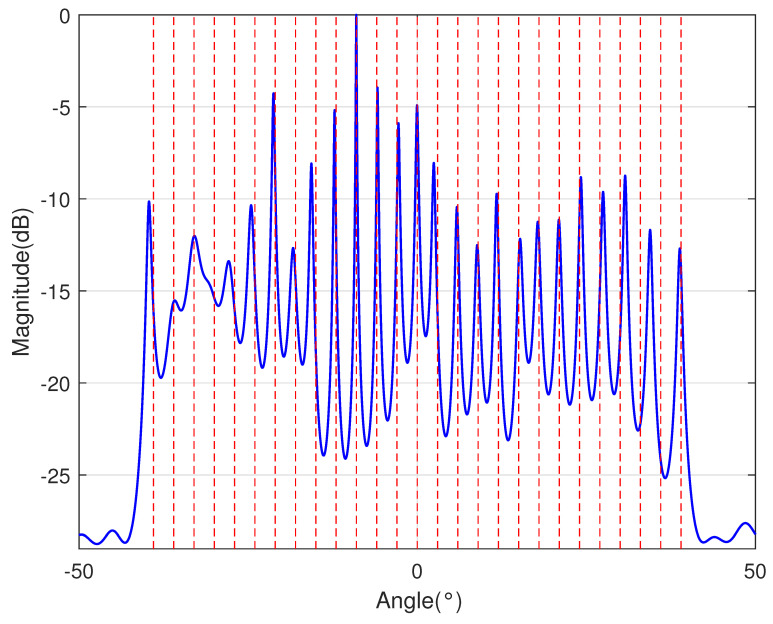	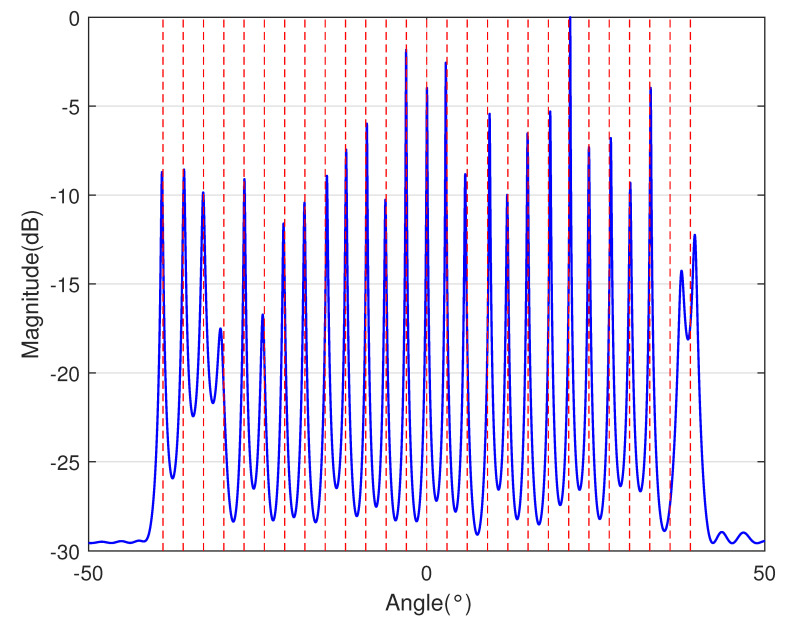	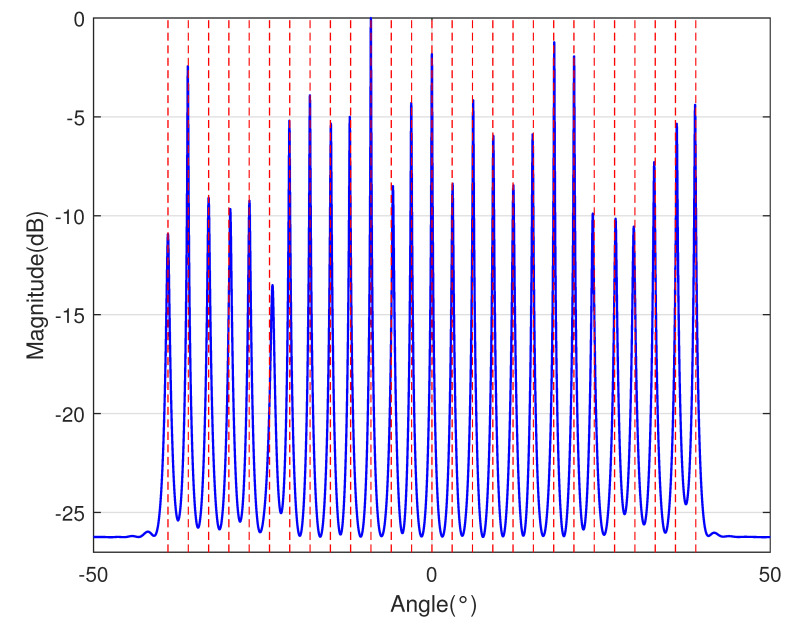
Consecutive DOFs	85	117	157
L(M)	0.4579	0.5438	0.3511
Normalized position	{0, 1, 2, 3, 4, 8, 12, 24, 36, 72}	{1, 2, 3, 4, 8, 12, 16, 32, 48, 64}	{0, 1, 2, 5, 8, 11, 23, 35, 71, 107}
2-SC	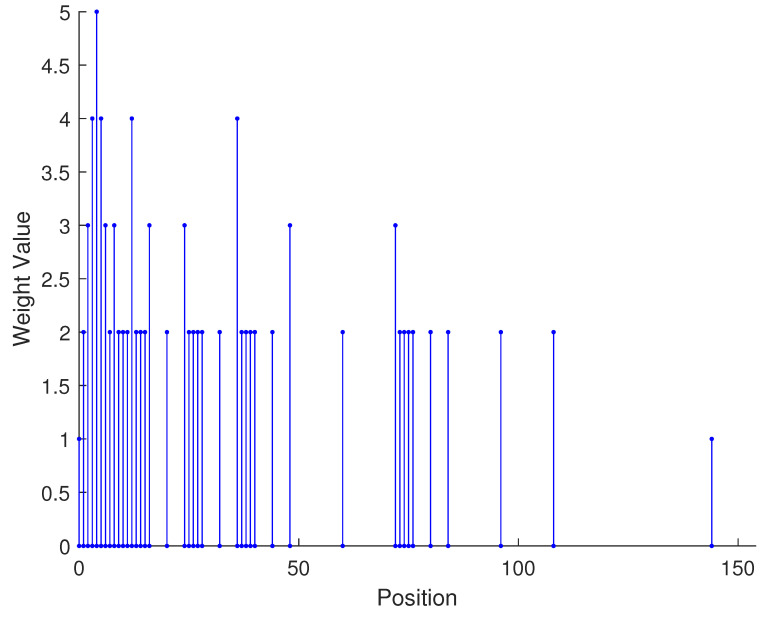	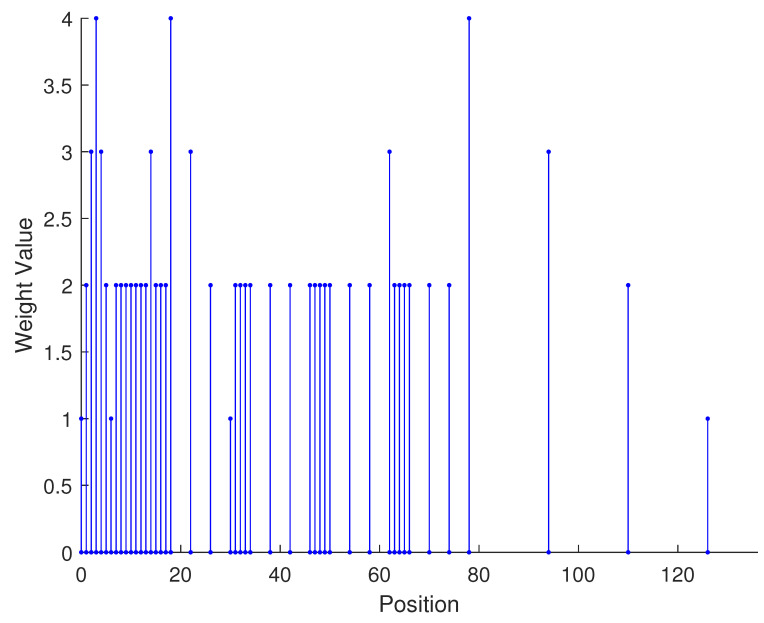	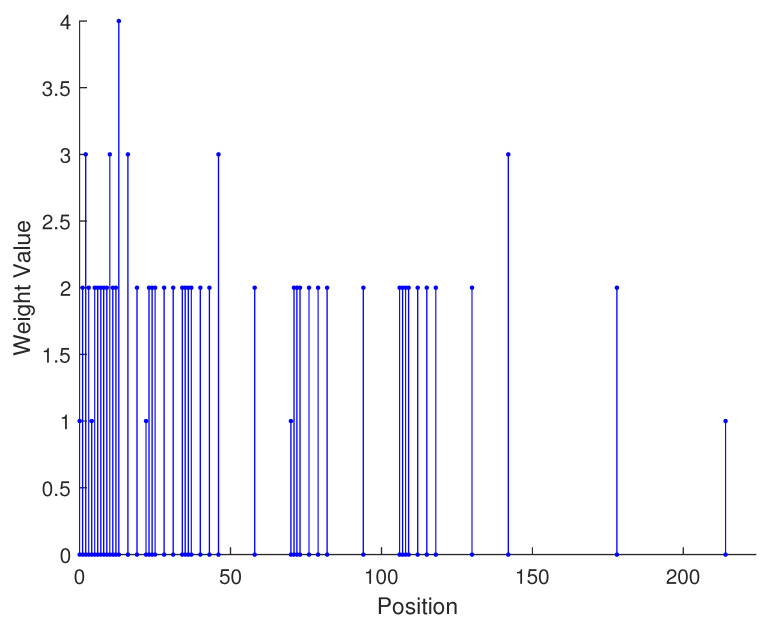
2-DCSC	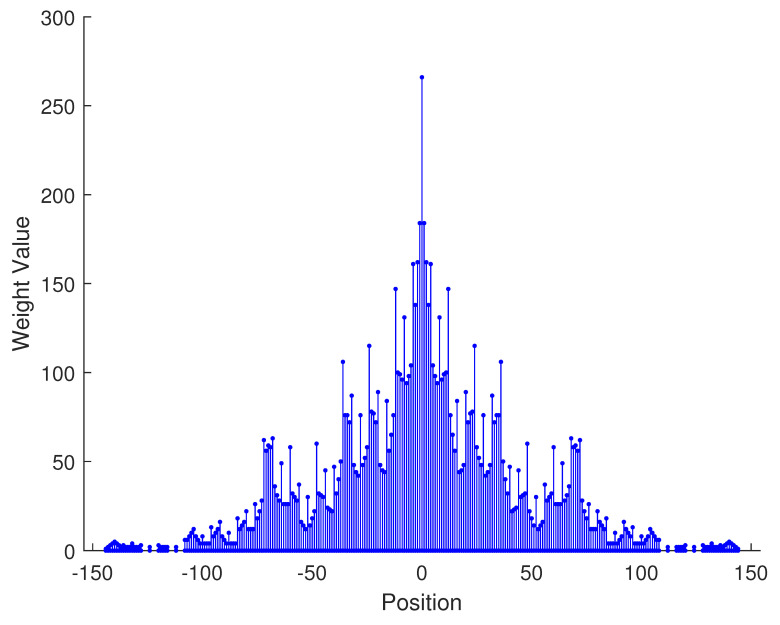	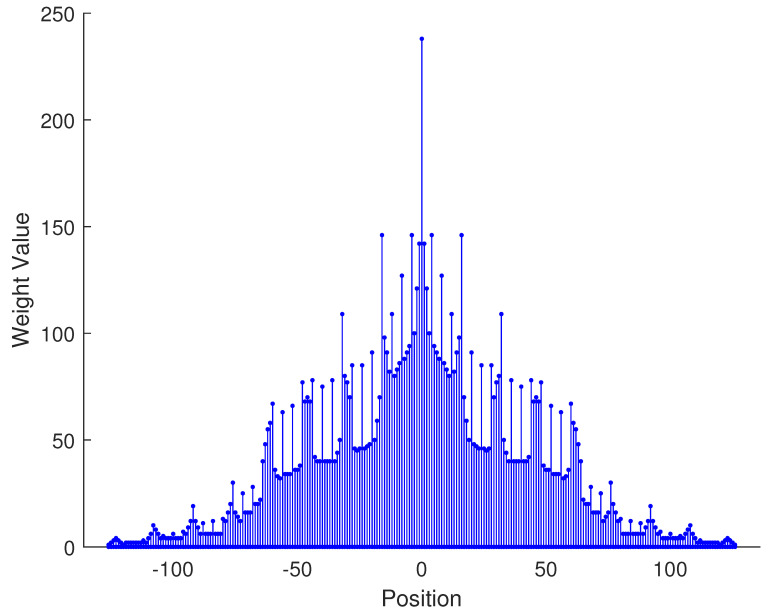	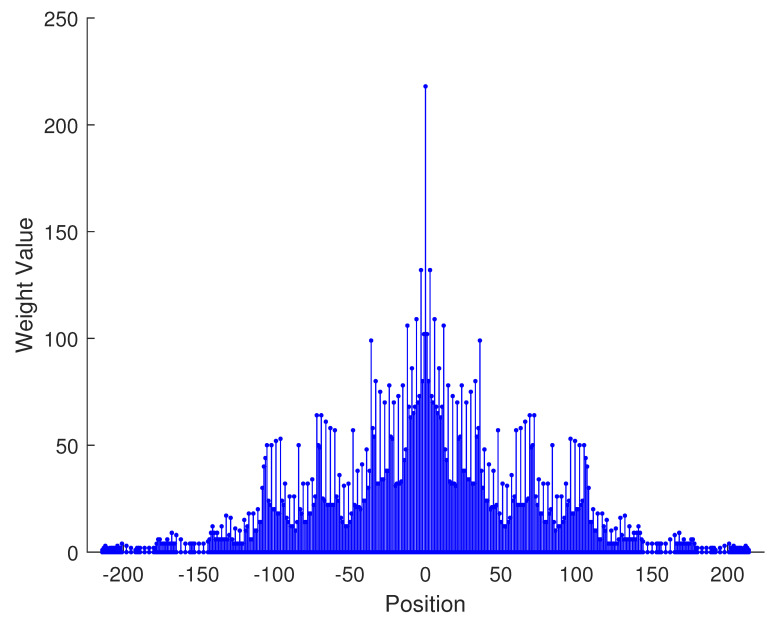
SS-MUSIC Spectrum	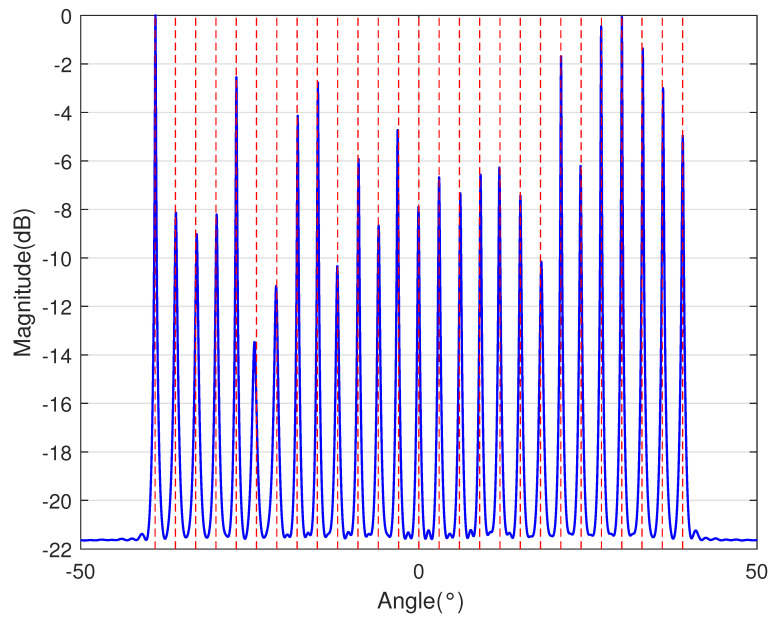	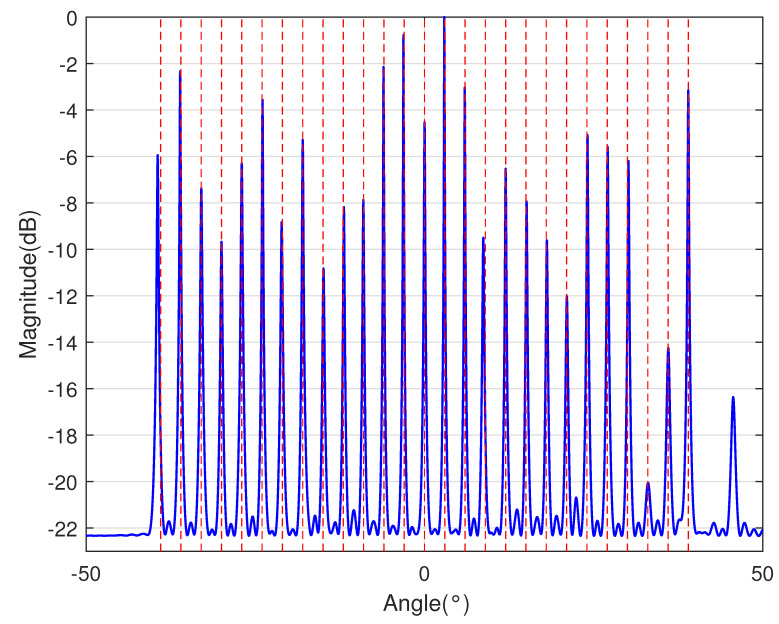	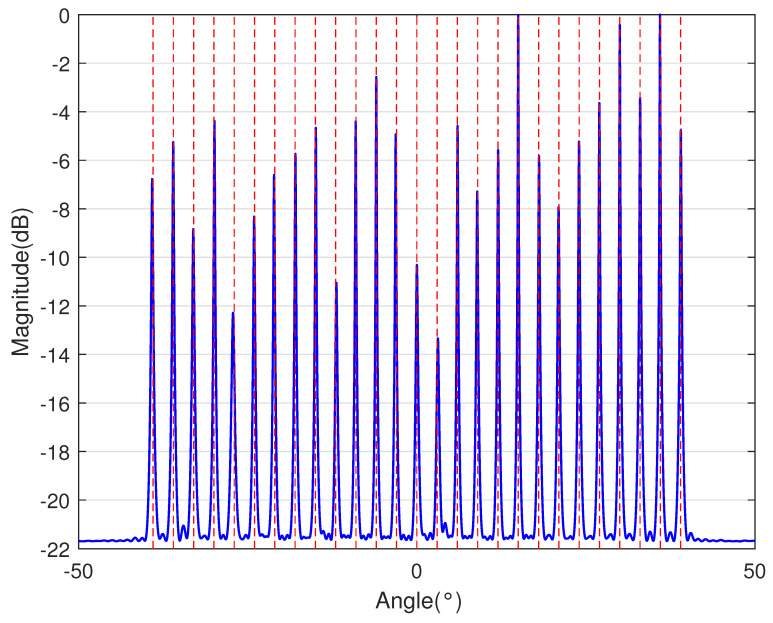
Consecutive DOFs	215	253	285
L(M)	0.5151	0.4819	0.4389

## Data Availability

Data are contained within the article.
